# Enhanced efficacy of oral hygiene instructions during motivational interviewing using intraoral scanner technology: a randomized clinical trial

**DOI:** 10.1007/s00784-026-06867-y

**Published:** 2026-04-23

**Authors:** Jacopo Lanzetti, Federica Ferrati, Armando Crupi, Umberto Gibello, Andrea Roccuzzo, Francesco Pera

**Affiliations:** 1 Department of Surgical Sciences - Dental School, University of Turin, Turin, Italy; 2 DIMEAS, Politecnico di Torino, Turin, Italy; 3 Shanghai Perio-Implant Innovation Center, Institute of Integrated Oral, Craniofacial and Sensory Research, Shanghai Ninth People’s Hospital, College of Stomatology, National Center for Stomatology, Shanghai Key Laboratory of Stomatology, Shanghai Jiao Tong University School of Medicine, Shanghai Jiao Tong University, National Clinical Research Center for Oral Diseases, Shanghai Research Institute of Stomatology, Shanghai, China; 4 Department of Periodontology, School of Dental Medicine, University of Bern, Bern, Switzerland

**Keywords:** Intraoral scanner, Concordance, Oral Hygiene Instruction, Plaque Control

## Abstract

**Objectives:**

To investigate the efficacy of an intra-oral scanner (IOS) as adjunctive tool to perform oral hygiene instructions during motivational interviewing (MI).

**Methods:**

A randomized controlled clinical trial (RCT) was performed in patients diagnosed with periodontitis Stage I-II, Gade A-B. Full Mouth Plaque Score (FMPS) (primary outcome) and Full Mouth Bleeding Score (FMBS) were recorded ad baseline (T0). Consequently, test group was scanned with an IOS, a digital 3D model created and was used during MI including oral hygiene instructions (OHI), while control patients received MI only. After *≥* 66 days (mean: 72 days) (T1), FMPS and FMBS as well as patients’ level of adherence and concordance by means of dedicated questionnaires were assessed and compared to T0.

**Results:**

A total of 80 patients (mean age 58.2 ± 15.2; 47 female; 62 non-smokers; 18 light smokers) were randomized and available for analysis. Mean FMPS change was 16.65 ± 22.95 for the test group and 6.98 ± 19.41 for the control group (*p* = 0.045), while FMBS change was 6.77 ± 7.30 for the test group and was 4.64 ± 12.87 for the control group (*p* = 0.366). Time for MI was 24.02 ± 1.60 min (Test group) and 22.52 ± 1.79 (Control group) (*p* < 0.001). Patients’ adherence, recorded as interdental care, showed a statistically significant difference in favor of the test group (test group: “yes”=37 (92.5%); “no”=3 (7.5%); control group: “yes”=23 (57.5%); “no”=17 (42.5%) (p < 0.001)).

**Conclusion:**

Within the limits of this RCT, the implementation of IOS to generate digital three-dimensional models of patients’ dentition demonstrated potential for improving the effectiveness of OHI during a single-session MI in terms of FMPS reduction.

**Clinical significance:**

The incorporation of intraoral scanning technology may represent a beneficial visual adjunct that strengthens patient-clinician communication and could contribute to enhanced self-performed oral hygiene, improved plaque control, and increase patient adherence.

**Supplementary Information:**

The online version contains supplementary material available at https://doi.org/10.1007/s00784-026-06867-y.

## Introduction

Most scientific evidence underlines the importance of proper domiciliary self-performed oral hygiene to prevent the onset of the most common oral pathologies [1–3]. Previous evidence by Van der Weijden and Slot DE showed that a single brushing procedure performed with a manual toothbrush can remove up to 42% of plaque, while the percentage of plaque removal increases to 46% if an electric toothbrush is used [4]. In addition, it has been pointed out the importance of performing interproximal plaque control, using the most suitable devices [5]. However, plaque removal is primarily related to patient’s adherence to self-performed oral hygiene [6]. In this context, patient’s behavioral change represents an important aspect that can hardly influence periodontal and implant therapy [7]. Communication with patients and motivational interviewing (MI) are crucial boost changing behavioral changes and improving patients’ adherence [8].

According to Miller and Rollnick [9], MI is a collaborative, goal-oriented communication style designed to strengthen intrinsic motivation and commitment to change. A systematic review, published in 2017, evaluated the effect of the MI, in addition to periodontal therapy, through clinical periodontal parameters and psychological factors related to oral hygiene [10] concluding that the MI would have a beneficial effect, but further research was needed to confirm this conclusion. The same conclusions have been reported by other authors [11, 12] that highlight how most studies on MI presented limits, such as short duration or low number of sessions (mostly only one). Other studies [13–15] have shown that repeating oral hygiene instructions is important in plaque index reduction. All quoted studies recognized the effectiveness of the MI, but there is no qualitative assessment of concordance, and adherence.

Routinely MI, often carried out using dental study cast models, has been shown to be not effective since patients cannot appreciate their oral situation [16]. On this aspect, rates of plaque-induced gingivitis remain high [17] despite studies indicating that oral hygiene instructions are regularly provided. A study conducted by Public Health England found that in 2018, 80% of dental patients were trained in oral hygiene at home, however 53% of the participants in the study had gum inflammation [18], suggesting a lack of effectiveness of the MI. In addition, it should be mentioned that the high prevalence of gingivitis despite proper high oral hygiene can also rely on the hypothesis that diet seems to be a determining factor on how the gingiva reacts towards dental plaque [19–21].

New technologies can be used as visual means of communication to implement patient-healthcare professional communication and improve concordance, and adherence [22]. Intraoral scanners (IOS), originally used for the design/manufacture of prosthetic restorations, are now used in a growing number of other applications [23, 24]. Over time, new generations of scanners have improved in accuracy, color reproduction [25] and can be used as visual support and aid to traditional communication approaches [26, 27]. IOS can create personalized digital dental models and highlight the patient’s gingival and hygienic status more effectively by showing plaque areas as a digital image on a monitor, stimulating the motivation and concordance of patients [28]. This suggests that the use of the IOS during MI could improve patient adherence to the instructions for home oral hygiene.

Hence, the aim of this study was to determine the potential adjunctive efficacy of the use of the IOS and the consequent creation of a digital model to a traditional MI as well as the evaluation of patient’s adherence to self-performed oral hygiene instructions. The null hypothesis was no statistically significant difference in Full Mouth Plaque Score (FMPS) (primary outcome measure) between the two interventions.

## Materials and methods

The study was approved by the Ethics Committee of “A.O.U. Città della Salute e della Scienza” of Turin (ref. 0000620) and was registered on ClinicalTrial.gov (NCT06904963). The study was conducted according to good clinical practice and the 2018 revised Declaration of Helsinki. Written informed consent was obtained from all participants prior to initiation of the study.

### Study design, group allocation and randomization

This was a parallel, single-center, randomized (1:1 ratio), controlled clinical trial conducted between January and May 2025 at the Department of Oral Rehabilitation, Maxillo-Facial Prosthesis and Implantology - C.I.R. Dental School, Turin, Italy.

**Fig. 1:**
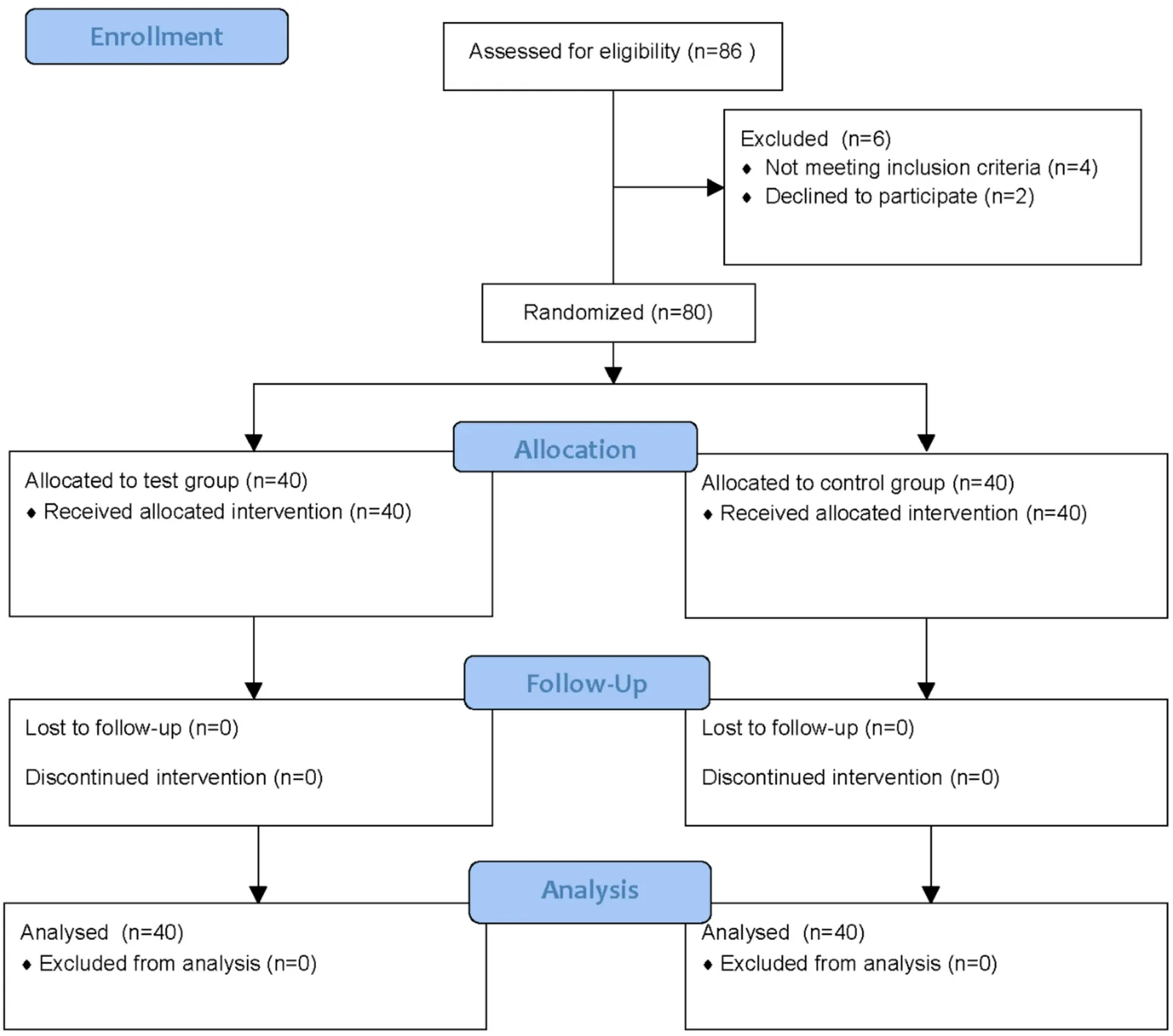
Study flowchart

The study flow chart is reported in Fig. 1. Data reporting followed the Consolidated Standards of Reporting (CONSORT) guidelines (Supplementary File 1). Patients were randomly allocated to the test (i.e., MI with intraoral scanner) and control (i.e., MI without intraoral scanner) group in accordance to randomization tables, while treatment allocation was concealed by using opaque envelopes which were labelled with the patient study number and opened immediately after baseline assessment and inclusion in the study by an external investigator (F.P.) not involved in the intervention or in the outcomes evaluation.

### Study population

Subjects referred to the Department of Oral Rehabilitation, Maxillo-Facial Prosthesis and Implantology - C.I.R. Dental School, Turin, Italy were consecutively screened for recruitment between January and February 2025. One investigator with 15 years of clinical experience (F.P.) evaluated all subjects and was responsible for patients’ enrollment following the assessment of inclusion and exclusion criteria.

#### Inclusion criteria

- Patients aged 18–80 years;
- Patients diagnosed with periodontitis stage I-II/grade A-B according to European Federation of Periodontology [29];
- Patients without systemic disease;
- Pregnancy and breastfeeding.

#### Exclusion criteria

- Patients with periodontitis stage III/IV;
- Patients with periodontitis grade C;
- Patients rehabilitated with dental implants affected by peri-implant diseases (mucositis and/or peri-implantitis) [29];
- Patients who have not undergone a professional oral hygiene session for more than 2 years;
- Patients receiving anti-inflammatory, antimicrobial or statin therapy;
- Immunocompromised patients;
- Smoking of more than 5 cigarettes per day, including electronic cigarettes;
- Patients with cognitive, manual and sensory deficits;
- Patients unwilling to attend.

### Intervention

Test group patients were scanned by an experienced operator (F.F.) in according to a previous study [30]. A 3D IOS 3D Opera Mach 2 (Opera system, Monaco) was used with the software Intelligent Scan version 3.3.2.10 (Opera system, Monaco) to create a digital 3D dental model of the patient mouth. Scanning procedure was carried out, after coloring dental plaque, with the use of a labial retractor (Optragate, Ivoclar Vivadent, Liechtestein) and the scanning area was kept dry by sucking saliva and using an air/water syringe. The scanning operator ensured that areas not sufficiently displayed were re-scanned until the entire dental arch was fully displayed. Thereafter, the 3D intraoral scan was saved as an STL-file, and the derived digital model was used for the patient’s during the MI. More specifically, the approach proposed and validated by Kopp et al. was followed [10]. In addition to this approach, personalized home oral hygiene instructions were provided, based on patient’s oral condition, to achieve optimal plaque control, according to previously validated tailored motivational approaches [31, 32]. More into details, the first part of the interview consisted in the explanation of need for good oral hygiene, a detailed instruction and demonstration of a powered toothbrush and the use of inter-dental brushes. The whole procedure was recommended to be performed twice per day for 2 min [3]. On the other hand, patients in the control group were instructed to domiciliary oral hygiene procedure through a traditional MI with the use of a mirror to explain the use of oral care devices directly in the patient’s mouth, according to the EFP Clinical Practice Guidelines [3]. All information and recommendations were discussed with the patient in an equivalent way, following the same order sequence of the above-described information. All interviews were conducted by an experienced RDH (F.F.) with over 5 years of clinical experience on this topic. An external operator (A.C.), not involved in data collection and statistical analysis, was responsible for ensuring that the MI was performed according to the standards required for the study. All MI sessions were performed for at least 20 min according to previous studies [33, 34]. A stopwatch measured the time used for the MI. For the test group, the time required for scanning as well as the time used by the software to create the 3D model were not taken into consideration.

At the completion of the MI, a session of professional supra-gingival instrumentation was performed by the same operator (F.F.) on all patients with the use of ultrasonic, hand instruments, and glycine powder, with the aim of removing all plaque and calculus deposits from the dental surfaces. At this stage, only Step 1 (including supragingival instrumentation), without the prescription of any antiseptics/antimicrobials rinse, was performed [3]. Following the end of the study, patients underwent an additional full chart periodontal examination and whenever a subject was found in need of additional therapy (i.e., Step 2), such treatment was explained and offer outside the present trial.

### Outcome measures

In accordance with a previous study [30], the primary outcome measure was plaque accumulation on the dental surfaces of the entire mouth expressed as Full Mouth Plaque Score (FMPS) [35]. The level of periodontal inflammation was evaluated by means of Full Mouth Bleeding Score (FMBS) [36] (secondary outcome measure).

The FMPS and the FMBS were recorded, assessing the presence or absence of plaque and bleeding on six surfaces of each tooth or dental implant [37]. The visual examination was performed under standardized conditions with standardized illumination and using a dental mirror and an XP23/UNC 15 periodontal probe (Hu-Friedy, Chicago, IL, USA). The FMPS was assessed using dental plaque staining by means of a cotton ball soaked in an erythrosin-based plaque detector (Mira-2-Ton Solution, Hager & Werken GmbH & Co., Duisburg, Germany). Subsequently, patients rinsed with water to eliminate detector excess. All examinations, including the one for periodontal diagnosis, were performed under 2.5x magnification by the same experienced and calibrated dental hygienist (J.L.) blinded to group allocation (Kappa score 0.908; 95% confidence interval [CI] 0.894 to 0.998; *P* < 0.01).

The assessment at T1 was performed according to a previously validated methodology: specifically, it took place after at least 66 days following the first session (T0) (i.e., median time for which 95% of new habits, in this case new devices or techniques of oral hygiene in the home, become routine) [38]. In this way, the patient was given the necessary time to learn the techniques and familiarize with the new aids that were recommended at T0. Moreover, at T1, the operator assessed the level of the patient’s adherence rating from 1 (no adherence: the patient has not adopted the detailed information provided during the previous session) to 5 (total adherence: the patient has successfully adopted all the detailed information provided in the previous session). Patient’s adherence to home hygiene procedures was evaluated both for brushing (i.e., type of brush and technique) and interdental hygiene (i.e., type of interdental care, technique and frequency) [39]. Scores from 1 to 5 were then dichotomized into “yes/no” classification: scores from 4 to 5 were considered “yes,” while scores from 1 to 3 were considered “no”. Furthermore, patients were asked to complete a short questionnaire to evaluate the level of concordance (i.e., the level of collaboration with the healthcare professional in making decisions on treatment) which emphasizes understanding, agreement and cooperation between the patient and healthcare on the treatment plan [40]. The questionnaire, proposed by the authors of this study, was structured on a Likert scale and was filled out by the patient independently and anonymously. Prior to compilation, the patient was asked to complete and asked to answer each question with a score from 0 (no improvement) to 5 (obvious improvement).

The questions included in the questionnaire were as follows:

1. Do you think your oral hygiene has improved since the given detailed information?
2. After the provided detailed information, can you understand the anatomical structures of your mouth better and where the plaque accumulates most?
3. After the provided detailed information, do you understand the use of the recommended home oral hygiene devices better?

### Statistical analysis

The sample size was calculated based on a previous study [30] and based on plaque accumulation on the dental surfaces (FMPS), with a confidence level of 95%, a type I error of 5% and an effecting size of 0,63. Therefore, 40 patients were required for each group, 80 patients in total. Quantitative variables were reported as mean ± standard deviation, while qualitative variables were reported as percentage. The Shapiro-Wilk test showed a normal distribution for the clinical variables (FMPS, FMBS) and for the adherence outcome. The distribution of concordance data was found to be non-normal. For the verification of the hypotheses, it was carried out respectively with the Student T-test for independent samples and with the Chi squared test. The Mann-Whitney U test was used for comparison between groups, for concordance data. Binomial logistic regression analysis was perormed. The statistical analysis was carried out by a blinded operator (U.G.) with respect to the provided treatment with a dedicated software (Jamovi - version 2.3.28.0).

## Results

From the original 86 enrolled patients, 80 (mean age 58.2 ± 15.2, 47 female and 33 male, 62 non-smokers, 18 light smokers (i.e., *≤* 5 cigarettes/day)) were randomized in two groups. Baseline patients’ characteristics did not differ between groups (*p* > 0.05). T1 assessment was performed after a mean period of 72 days (Min 66 - Max 96; *p* = 0.250). Details of patients’ demographic and clinical data at T0 are shown in Table 1 while the study flow chart is shown in Fig. 3.

**Table 1:** Baseline (T0) demographic characteristics and clinical data of test and control group

	Test (*N* = 40)	Control (*N* = 40)	Total (*N* = 80)	*p*-value
Gender				0.256
Female	26.0 (65.0%)	21.0 (52.5%)	47.0 (58.8%)	
Male	14.0 (35.0%)	19.0 (47.5%)	33.0 (41.2%)	
Age				0.367
Mean ± SD	59.7 ± 13.9	56.6 ± 16.5	58.2 ± 15.2	
Smoking status				0.592
Yes	10.0 (25.0%)	8.0 (20.0%)	18.0 (22.5%)	
No	30.0 (75.0%)	32.0 (80.0%)	62.0 (77.5%)	
Education level				0.431
Primary/secondary education	11.0 (27.5%)	8.0 (20.0%)	61.0 (76.2%)	
Bachelor/master or higher education	29.0 (72.5%)	32.0 (80.0%)	19.0 (23.8%)	
Periodontal Diagnosis				0.748
Stage I Grade A	19.0 (47.5%)	19.0 (47.5%)	38.0 (47.5%)	
Stage I Grade B	13.0 (32.5%)	15.0 (37.5%)	28.0 (35.0%)	
Stage II Grade A	1.0 (2.5%)	0.0 (0.0%)	1.0 (1.2%)	
Stage II Grade B	7.0 (17.5%)	6.0 (15.0%)	13.0 (16.2%)	
N° Teeth				0.071
Mean ± SD	27.5 ± 1.3	28.1 ± 1.6	27.8 ± 1.5	
N° Implant				0.887
Mean ± SD	0.5 ± 0.6	0.5 ± 0.9	0.5 ± 0.8	
Probing Depth (PD)				0.799
Mean ± SD	2.0 ± 0.4	2.1 ± 0.4	2.0 ± 0.4	
Clinical Attachment Level (CAL)				0.847
Mean ± SD	2.1 ± 0.4	2.1 ± 0.4	2.1 ± 0.4	
FMPS_T0				0.077
Mean ± SD	46.6 ± 21.0	38.5 ± 19.3	42.6 ± 20.4	
FMBS_T0				0.770
Mean ± SD	13.7 ± 9.6	14.4 ± 13.5	14.0 ± 11.6	
Time from OH session				0.658
Mean ± SD	3.0 ± 0.5	3.1 ± 0.5	3.0 ± 0.5	

Note: Mean ± SD; Number (percentage). Pearson’s Chi-squared test and Linear Model ANOVA were used for between groups comparisons

Abbreviations: FMPS, Full Mouth Plaque Score (%); FMBS, Full Mouth Bleeding Score (%); OH: Oral Hygiene

FMPS (primary outcome measure) decreased from 46.61 ± 20.96 to 29.96 ± 17.79 in test group and from 38.53 ± 40.74 to 31.55 ± 17.42 in the control group. FMPS change was 16.65 ± 22.95 for the test group and was 6.98 ± 19.41 for the control group. This difference was statistically significant (*p* = 0.045). With respect to secondary outcome, FMBS decreased from 13.65 ± 9.56 to 6.88 ± 6.34 in test group and from 14.42 ± 13.47 to 9.78 ± 7.78 in the control group. This led to a change of 6.77 ± 7.30 for the test group and was 4.64 ± 12.87 for the control group. This difference was not statistically significant (*p* = 0.366).

The calculated time for MI was 24.02 ± 1.60 min for the test group and was 22.52 ± 1.79 min for the control group. This difference was statistically significant (*p* < 0.001). Details of the quantitative variables are shown in Table 2.

**Table 2:** FMPS and FMBS at T0 and T1 within the test and control group

	Baseline (T0)			Follow-up (T1)						
	Test group (*N* = 40)	Control group (*N* = 40)	*p*-value	Test group (*N* = 40)	Control group (*N* = 40)	*p*-value		Change T1-T0 Test group (*N* = 40)	Change T1-T0 Control group (*N* = 40)	*p*-value
FMPS	46.61 ± 20.96 (39.9–53.3)	38.53 ± 40.74 (32.3–44.7)	0.077	29.96 ± 17.79 (24.3–35.7)	31.55 ± 17.42 (26.0–37.1.0.1)	0.687		16.65 ± 22.95 (9.31–24.0.31.0)	6.98 ± 19.41 (0.77–13.2)	**0.045**
FMBS	13.65 ± 9.56 (10.6–16.7)	14.42 ± 13.47 (10.1–18.7)	0.770	6.88 ± 6.34 (4.86–8.91)	9.78 ± 7.78 (7.29–12.3)	0.072		6.77 ± 7.30 (4.44–9.10)	4.64 ± 12.87 (0.53–8.76)	0.366
Time	24.02 ± 1.60	22.52 ± 1.79	< 0.001	NA	NA	NA		NA	NA	NA

Note: Mean ± SD (95%CI). Student t test were used for between groups comparisons

Abbreviations: FMPS, Full Mouth Plaque Score (%); FMBS, Full Mouth Bleeding Score (%); Time, time for motivational interview (minutes)

Patients’ adherence recorded as brushing technique and interdental care showed a statistically significant difference in favor of the test group with respect to the second variable only (test group: “yes”=37 (92.5%); “no”=3 (7.5%); control group: “yes”=23 (57.5%); “no”=17 (42.5%) (p < 0.001)) (Table 3).

**Table 3:** Comparison of adherence to brushing and interdental hygiene in the two groups at T1

	Test group (*N* = 40)	Control group (*N* = 40)	Total	*p*-value
Brushing adherence				
Yes	35 (87.5%)	36 (90.0%)	71 (88.75%)	
No	5 (12.5)	4 (10.0%)	9 (11.25%)	0.723
Interdental hygiene adherence				
Yes	37 (92.5%)	23 (57.5%)	60 (75.0%)	
No	3 (7.5%)	17 (42.5%)	20 (25.0%)	**< 0.001**

Note: Number (percentage). Chi-squared test were used for between groups comparisons

With respect to assessment of patient’s concordance, no statistically significance differences were detected (*p* > 0.05) despite the average higher level of concordance noticed in the test group. Details of the concordance scores are shown in Table 4.

**Table 4:** Level of concordance of patients in the two groups at T1

Questions	Group	*N*	Mean ± SD	*p*-value
1. Improving oral hygiene at home	Test	40	3.67 ± 1.27	
	Control	40	3.27 ± 1.72	0.487
2. Self-assessment plaque	Test	40	4.15 ± 1.05	
	Control	40	3.60 ± 1.48	0.103
3. Understanding oral care devices	Test	40	4.33 ± 0.66	
	Control	40	3.77 ± 1.46	0.146

Note: Mean ± SD. U Mann-Whitney test were used for between groups comparisons

Finally, the logistic regression analysis did not reveal any significant effect of the investigated variables on the outcome with respect to the groups (Table S1-S2).

## Discussion

**Fig. 2:**
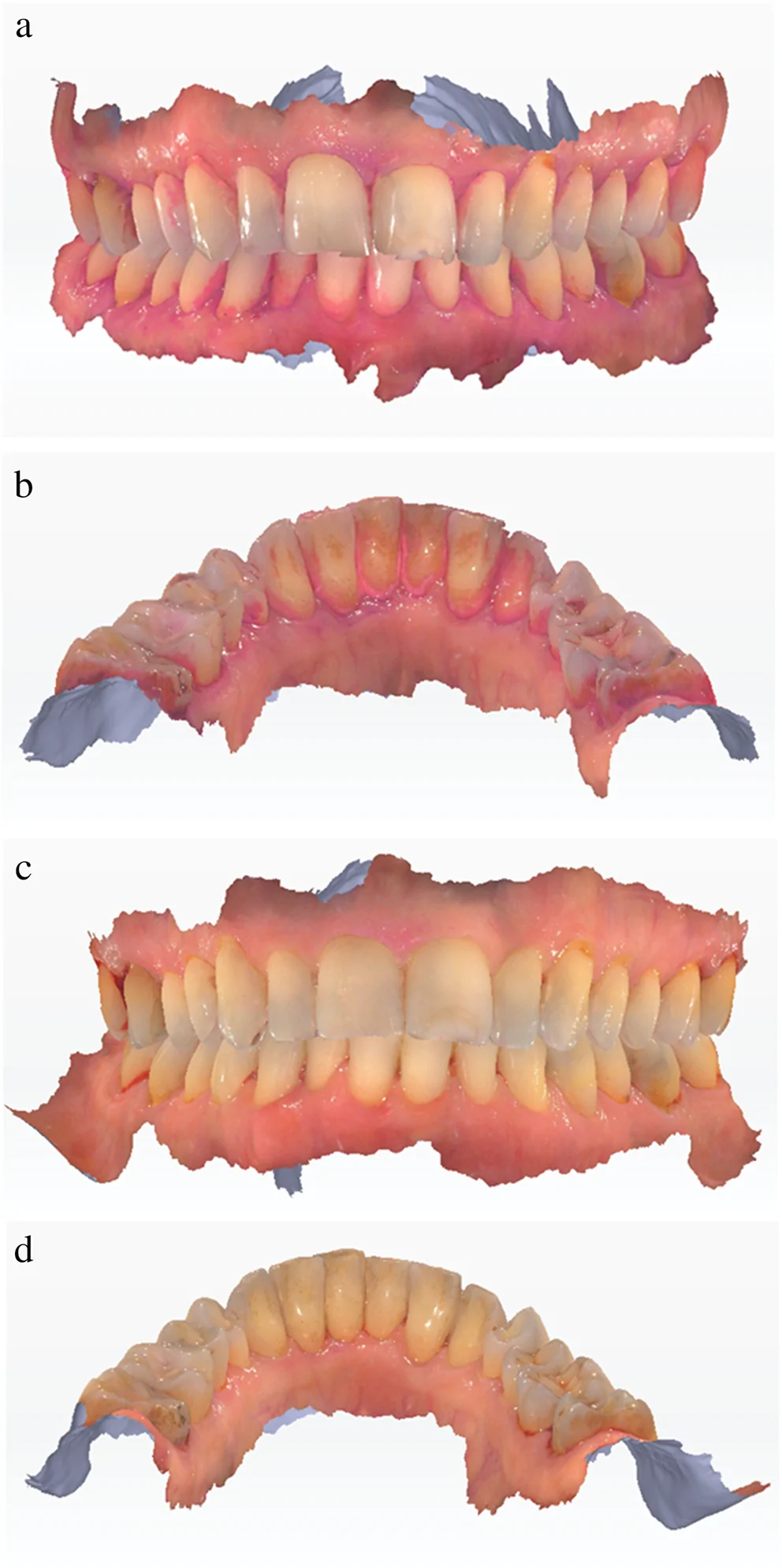
Representative digital scenario of a test patient at T0: frontal (**a**) and lingual view (**b**); and at T1: frontal (**c**) and lingual view (**d**)

**Fig. 3:**
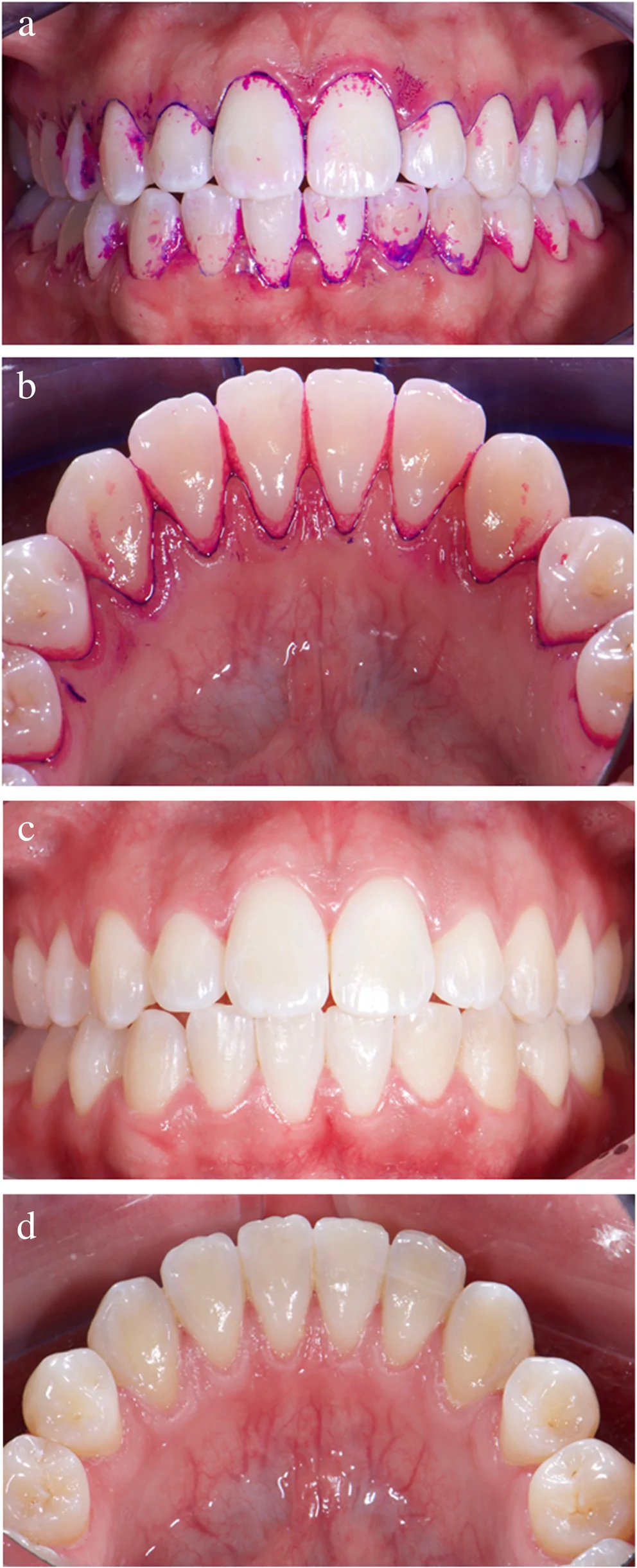
Representative digital scenario of a control patient at T0: frontal (**a**) and lingual view (**b**); and at T1: frontal (**c**) and lingual view (**d**)

This study aimed to evaluate the efficacy of a single session MI conducted with the aid of a 3D digital model, obtained by scanning patient’s dental arches. Based on the obtained results, after a mean follow-up of 72 days, it can be stated that MI conducted with the aid of a digital scan was effective in reducing plaque levels (Figs. 2 and 3).

Although plaque and bleeding indices improved in both groups, the MI performed with the IOS has been shown to significantly improve clinical plaque scores compared to the motivation performed the traditional way. These results are in accordance with previous investigations highlighting how the intraoral scanners are an effective tool to improve patient’s understanding of diagnosis and treatment planning [30, 41] and more specifically for improving patient education and motivation, and therefore communication with patients in general [42].

The use of IOS for dental plaque examination could be useful as a new method for dental hygiene education, as confirmed by other authors [43]. In particular, patients may have found it easier to visualize oral problems and better understood oral hygiene recommendations when explained through the use of intraoral scans [44].

The MI carried out with the scanner led to greater plaque reduction, however, the same result did not occur for the FMBS. This finding might be interpreted as the consequence of the short follow-up period as underlined in another study [30]. Furthermore, a study that used an intraoral camera to instruct patients showed significant differences in bleeding levels after 4 months [45]. In addition, it should be noticed that all included patients already had very low FMBS (< 20%) at baseline.

Regarding the time needed to provide oral hygiene instructions, it can be stated that the time used in our study is consistent with other studies [33, 34]. More specifically, it should be noticed that the overall time was significantly longer in the test group: this is significant because the operator dedicates more time to explaining to the patient how to improve their oral hygiene at home, since the digital model is a more effective means of communication than the mirror, traditionally used for motivational interviewing. In fact, even though the mirror can detect plaque on the labial and buccal surfaces, the lingual or palatal surfaces are difficult for the patient to see. Intraoral scanning makes it possible to obtain a 3D image that can be easily rotated and manipulated by software and viewed from all angles. With 3D imaging, plaque can be detected even on surfaces that are normally challenging to be observed, such as interdental surfaces, and this allows a clearer explanation of the oral hygiene procedures that the patient should implement. Consequently, MI was more comprehensive and more complete.

Adherence to oral hygiene instructions assessed by patient self-declaration improved in both groups. Specifically, while adherence to the brushing techniques did not show differences between groups, patients in the test group showed statistically higher adherence to oral hygiene tips. This may suggest that the use of a 3D model from a dental arch scan can provide a clear and useful reference for the patient to better understand sites that require greater attention to oral hygiene, such as interdental sites [42], whereas the instructions on brushing techniques, being easier to learn, are not influenced by the MI modality. In this case, the use of the scanner and the 3D digital model of the patient’s arches increases the patient’s adherence to self-performed oral hygiene because it improves the patient’s knowledge of their own mouth [26, 27].

When focusing on the factors potentially affecting interdental adherence, it should be underlined that the detected improvement in the test group was not influenced by patients’ sex, as well as by other potential confounders (i.e., smoking status; educational level). These findings suggest that the efficacy of the tested method is robust and independent of the primary demographic and behavioral characteristics of the sample. Although the complex model showed high standard errors for certain interaction terms, the overall evidence supports the broad applicability of the intervention across different patient profiles. In addition, the logistic regression analysis did not reveal a significant effect in the interaction between baseline plaque levels and group, suggesting that the effectiveness of the intervention was not affected by initial participants’ OH status.

However, at the time being considering the exploratory nature of the present trial, no definitive conclusion on this aspect can be drawn.

From the analysis of concordance responses, we could notice a higher level of concordance in patients who have been instructed on home oral hygiene with the aid of the intraoral scanner; however, this difference was not statistically significant. Therefore, the use of a digital scan during MI encouraged patient’s collaboration and participation during the oral hygiene demonstrative procedures.

Although not statistically significant, patients who were instructed using a 3D digital model have a higher level of concordance than patients who received only verbal information with the aid of a mirror. The same result was achieved by a similar study where patients who the IOS treatment plan was explained to responded more positively than patients who received only a verbal explanation [41]. The advantage of using the IOS is that it can present oral conditions with greater specificity and in a very personalized way. This allows personalizing the communication with the patient who feels at the center of treatment plan and more involved in the oral hygiene instructions, thus increasing the concordance. In this study, using 3D scans of the dental arches, patients from the test group were able to clearly see areas where removal of dental plaque is harder. In this case, the use of the IOS facilitates the change of behavior regarding self-performed oral hygiene and has also proved to be an effective technique for improving oral health [19]. Scanning the oral cavity with a 3D scanner can help assess the state of oral hygiene. Moreover, it is a simple and easy technology to learn for the operator, without any complications for the patient [46].

The 3D image is enabled for magnification and rotation, which allows an easier evaluation of areas with less visibility. This is highlighted by a study that reported how digital intraoral monitors are considered more useful than dental mirrors for the evaluation of plaque and tartar [47] and offer an advantage in plaque detection thanks to their three-dimensional imaging capabilities [48]. In addition, 3D images can be easily archived, allowing evaluation by several operators.

However, scanning the arches may have some limitations. The size of the scan terminal can make it difficult to position when the mouth opening is limited. Also, the scan may be affected by the height of the masticatory muscles and the jaw crest. In addition, the time required to carry out the scan, if carried out only for the MI and not also for prosthetic or orthodontic reasons, is to be considered an additional time to the session.

Despite the randomized nature, this has some limitations. First, patients were not blinded to the provided treatment. Furthermore, provider-rated adherence, rated on a scale of 1 to 5 and consequently dichotomized for data analysis reasons, has been proposed but not previously validated. Second, the sample is recruited from a single university-setting. Third, the follow-up is short, and the obtained results are based on a single intervention, while it is common clinical experience that patients often require several sessions of oral hygiene instructions before showing of having properly incorporated oral hygiene procedures [49]. Fourth, no specific tool such as MITI or MI-SCOPE was used to assess the quality of the MI. Finally, home oral hygiene instructions were not standardized but tailored to the patient’s clinical condition, which could increase variability on self-performed oral hygiene procedures performed at home, especially in cases of advanced periodontitis (Step III and IV). One additional aspect which should be recalled it that, following MI, all patients received supragingival instrumentation in accordance with EFP clinical guidelines [3]; therefore, the improvement observed at T1 in terms of FMPS not only reflects the patients’ oral hygiene, but also includes professional procedures. However, since this procedure was performed in both groups, the observed difference reflects the additional positive effect of the studied procedure. Furthermore, a specific analysis of the primary outcome measure changes taking into consideration initial FMPS values was not performed. Finally, since patients affected by severe periodontitis and peri-implant diseases were excluded from the present study, the generalizability of the results obtained from this clinical scenario is limited and should be interpreted with caution.

## Conclusions

Within the limitations of this randomized clinical trial, the implementation of intraoral scanning systems to create digital three-dimensional models of patients’ dentition demonstrated potential for improving the effectiveness of OHI in the context of a in single-session MI. The incorporation of intraoral scanning technology may represent a beneficial visual adjunct that strengthens patient-clinician communication and could contribute to enhanced self-performed oral hygiene, improved plaque control, and increase patient adherence.

## Supplementary Information

Below is the link to the electronic supplementary material.

Supplementary Material 1 (DOCX 37.2 KB)

Supplementary Material 2 (DOCX 25.9 KB)

## Data Availability

The data that support the findings of this study are available upon reasonable request from the corresponding author. The data are not publicly available due to privacy or ethical restrictions.
