# Supplementary material for: Enhanced efficacy of oral hygiene instructions during motivational interviewing using intraoral scanner technology: a randomized clinical trial
Source: Clin Oral Investig. 2026 Apr 23;30(5):189. doi: 10.1007/s00784-026-06867-y (PMC13658496; doi:10.1007/s00784-026-06867-y)
Supplement: Supplementary file 2 [file 784_2026_6867_MOESM2_ESM.docx]

**Supplementary materials**

**Table S1.** Model Coefficients - Interdental Adherence, Gender, Smoking status and Educational level

| **Predictor** | | **Estimation** | | **SE** | | **Z** | | **p** | |
| --- | --- | --- | --- | --- | --- | --- | --- | --- | --- |
| Intercept |  | 0.485 |  | 0.496 |  | 0.97659 |  | 0.329 |  |
| Group: |  |  |  |  |  |  |  |  |  |
| Test – Control |  | 1.713 |  | 1.165 |  | 1.47010 |  | 0.142 |  |
| Gender: |  |  |  |  |  |  |  |  |  |
| Male – Female |  | -0.420 |  | 0.666 |  | -0.63056 |  | 0.528 |  |
| Smoking status: |  |  |  |  |  |  |  |  |  |
| Yes – No |  | 0.814 |  | 0.925 |  | 0.87984 |  | 0.379 |  |
| Educational level: |  |  |  |  |  |  |  |  |  |
| Primary, secondary education – Bacherol, master or higher |  | -0.442 |  | 0.705 |  | -0.62689 |  | 0.531 |  |
| Group ✻ Gender: |  |  |  |  |  |  |  |  |  |
| (Test – Control) ✻ (Male – Female) |  | -0.168 |  | 1.468 |  | -0.11453 |  | 0.909 |  |
| Group ✻ Smoking status: |  |  |  |  |  |  |  |  |  |
| (Test – Control) ✻ (Yes – No) |  | 16.205 |  | 3114.327 |  | 0.00520 |  | 0.996 |  |
| Group ✻ Educational level: |  |  |  |  |  |  |  |  |  |
| (Test – Control) ✻ (Up to high school – Higher than high school) |  | 17.598 |  | 2888.810 |  | 0.00609 |  | 0.995 |  |

**Table S2.** Model Coefficients - Interdental Adherence vs FMPS at baseline

| **Predictor** | | **Estimation** | | **SE** | | **Z** | | **p** | |
| --- | --- | --- | --- | --- | --- | --- | --- | --- | --- |
| Intercept |  | -0.1550 |  | 0.7235 |  | -0.214 |  | 0.830 |  |
| FMPS_T0 |  | 0.0120 |  | 0.0171 |  | 0.699 |  | 0.485 |  |
| Group: |  |  |  |  |  |  |  |  |  |
| Test – Control |  | 4.0771 |  | 1.9896 |  | 2.049 |  | 0.040 |  |
| FMPS_T0 ✻ Group: |  |  |  |  |  |  |  |  |  |
| FMPS_T0 ✻ (Test – Control) |  | -0.0394 |  | 0.0357 |  | -1.104 |  | 0.270 |  |
